# *Mycobacterium tuberculosis* infection may increase the degrees of malignancy in lung adenocarcinoma

**DOI:** 10.3389/fimmu.2025.1537520

**Published:** 2025-02-21

**Authors:** Shanshan Li, Mengru Feng, Fenghua Wang, Dongxu Liu, Mingyan Li, Jinlong Dai, Yan Yang, Yinghui Chai, Wen Chen

**Affiliations:** ^1^ Department of Pathology, The Eighth Medical Center, General Hospital of the Chinese People's Liberation Army (PLA), Beijing, China; ^2^ Department of Radiology, The Eighth Medical Center, General Hospital of the Chinese People's Liberation Army (PLA), Beijing, China; ^3^ Department of Clinical Laboratory, The Eighth Medical Center, General Hospital of the Chinese People's Liberation Army (PLA), Beijing, China

**Keywords:** *Mycobacterium tuberculosis*, lung cancer, immunotherapy, PD-L1, granuloma

## Abstract

**Background:**

The early diagnosis and management of lung adenocarcinoma co-existing with tuberculosis (LAC-TB) presents significant challenges in clinical settings. This is compounded by a paucity of robust clinical evidence elucidating the interactions between these two conditions.

**Methods:**

This study included 14 patients diagnosed with LAC-TB, with an equal distribution among those with pulmonary tuberculosis (TB) and those with peripheral lymph node TB. Controls included patients with simple TB and those with lung adenocarcinoma (LAC). Histopathologic examinations confirmed typical changes in each group. Immunohistochemistry analyzed immune markers, focusing on PD-L1, while genomic analysis identified differential mutant genes.

**Results:**

Pathological evaluations showed that LAC-TB and LAC groups expressed TTF-1 and Napsin A in their adenocarcinoma specimens. Notably, a higher proportion of patients in the LAC-TB group had a Ki-67 proliferation index of ≥10%. Subsequent Molecular analyses revealed significant differences in RALGAPA1 gene expression, with the LAC-TB group also exhibiting a greater median count of missense mutations, single nucleotide polymorphisms, and overall mutations, suggesting a higher malignancy level than the LAC group. Additionally, the LAC-TB group showed an increased tumor mutational burden, indicating a potentially better response to immunotherapy. Immunohistochemical assessments indicated that *Mycobacterium tuberculosis* (MTB) infection correlated with reduced infiltration of T cells and CD4^+^ T cells, alongside an upregulation of PD-L1 expression in LAC. Notably, PD-L1 was strongly expressed in the TB granuloma and surrounding areas.

**Conclusion:**

Our findings suggest that MTB infection may increase the malignancy of LAC, with the pronounced expression of PD-L1 in granuloma regions constituting a pivotal mechanism underlying this relationship.

## 1 Introduction

Globally, lung cancer (LC) is the most common malignant tumor and the leading cause of cancer-related death (1). Tuberculosis (TB), primarily caused by *Mycobacterium tuberculosis* (MTB), remains a significant public health issue, with pulmonary TB (hereafter referred to as TB) being the most prevalent manifestation (2, 3). Active TB is observed in approximately 2-5% of LC patients, while LC is found in 1-2% of those with active TB (4). The coexistence of TB and LC (LC-TB) complicates clinical diagnosis due to overlapping symptoms such as cough, fever, hemoptysis, and similar imaging findings on X-rays and computed tomography (CT) scans (5, 6).

Three main theories attempt to explain the coexistence of these diseases: TB as a predisposing factor for LC, the potential for anticancer therapies to reactivate latent TB, or a random association (7). Inflammation and infection are significantly linked to LC development; for instance, TB-induced airway inflammation elevates the risk of LC with a relative risk ratio of 1.36-1.90, independent of smoking status (8, 9). Chronic inflammation resulting from MTB infection contributes to recurrent lung tissue damage, facilitating LC pathogenesis, particularly lung adenocarcinoma (LAC) (10, 11). Moreover, TB is frequently encountered in advanced LC cases, heightening the risk of opportunistic infections due to immunosuppression (12). Treatments for LC, including systemic chemotherapy and immunotherapy, are intricately associated with TB management (13, 14).

Immunotherapy has transformed the therapeutic landscape for non-small cell lung cancer (NSCLC), improving patients’ survival and quality of life (15). Immune checkpoint inhibitors (ICIs), such as anti-programmed cell death protein 1 (PD-1) and anti-cytotoxic T lymphocyte-associated antigen-4 (CTLA-4) monoclonal antibodies, have shown efficacy across various treatment settings, including neoadjuvant and adjuvant therapies (16, 17). The PD-1/PD-L1 and CTLA-4 pathways represent prevailing strategies in LC immunotherapy (15). These agents seek to counter immune evasion mechanisms, highlighting the critical influence of the tumor microenvironment (TME), which comprises both tumor and non-tumor cells, including immune cells, on tumor dynamics and progression (18, 19).

Recent studies affirm the clinical benefit of concurrent anti-TB therapy with chemotherapy for patients with LC-TB (7, 20). However, there exists a concern regarding TB reactivation during anti-PD-1/PD-L1 immunotherapy in LC-TB patients. While immunotherapy is generally safe for individuals with latent TB, caution is warranted for those with active TB due to the risk of immune-related adverse events (irAEs) (21). Including patients with chronic infections like TB in clinical trials has been limited due to potential complications (22, 23). Screening for latent TB infection may be beneficial, and it is suggested that ICIs could represent a promising treatment avenue for LC patients with a history of TB disease. This study aims to explore the effects of MTB infection on LAC at both the histological and molecular levels, offering insights for early diagnosis and targeted treatments.

## 2 Materials and methods

### 2.1 Study subjects

A total of 48 patients were included in this study, all of whom underwent surgical treatment at the Eighth Medical Center of the Chinese People’s Liberation Army General Hospital and were confirmed by postoperative pathological examination. Patients had to meet the following inclusion criteria (1): Complete medical and clinical records, including age, gender, smoking history, TB history, tumor staging, diameter, histological type, and general condition (2). No prior anti-TB treatment, and no previous surgeries or therapies related to tumors, including chemotherapy, immunotherapy, or the use of immunostimulants or immunosuppressants (3). No history of other malignant tumors or immune system diseases (4). Pathology specimens must be formalin-fixed, paraffin-embedded, and H&E stained, confirmed by pathologists to show typical granulomatous inflammation and caseous necrotic lesions for TB, as well as tumor cell features for LAC. The paraffin blocks should meet quality standards and be suitable for immunohistochemical staining. The exclusion criteria included autoimmune disease, malignancy other than LAC, hepatitis B or C infection, HIV infection, serious fungal or bacterial infections, or serious hematological disorders.

### 2.2 H&E staining

Tissue samples were sliced into 3 µm thick sections and mounted on slides. After baking at 72°C for 30 minutes, the sections were deparaffinized with xylene and graded ethanol. They were stained with hematoxylin for 3 minutes, differentiated in hydrochloric acid, counterstained with ammonia, and restained with eosin. Finally, the sections were dehydrated in ethanol, cleared in xylene, and sealed with neutral resin.

### 2.3 Acid-fast staining

Tissue samples were cut into 3 µm thick sections and attached to slides. They were baked at 72°C for 30 minutes, then deparaffinized in xylene and graded ethanol. The slides were stained with carbonate fuchsin for 2 hours, decolorized with hydrochloric acid alcohol, and counterstained with hematoxylin for 30 seconds. After differentiation with hydrochloric acid and bluing with ammonia water, they were dehydrated in graded ethanol, cleared in xylene, and mounted with neutral resin.

### 2.4 *Mycobacterium* species identification

**Figure 1:**
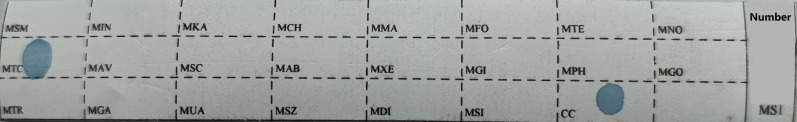
Sequence of probes on membrane strips. MTC, *Mycobacterium tuberculosis complex*; CC, quality control loci; The rest are non-*Mycobacterium tuberculosis* detection loci.

Ten tissue sections, each 5 µm thick, were placed in 1.5 mL centrifuge tubes and underwent deparaffinization, lysis, and enzymatic digestion to extract DNA. Four microliters of this DNA were added to PCR tubes from the *Mycobacterium* species identification gene test kit (Ya Neng Biotechnology Co., Ltd., Shenzhen, China). The amplification procedure began with 50°C for 2 minutes and 95°C for 10 minutes, followed by 30 cycles of 95°C for 45 seconds and 68°C for 60 seconds, and another 30 cycles of 95°C for 30 seconds, 54°C for 30 seconds, and 68°C for 60 seconds, ending with a final extension at 68°C for 10 minutes. After amplification, the products and membranes were placed in solution A (890 mL distilled water, 100 mL 20× sodium citrate, 10 mL 10% sodium dodecyl sulfate) and heated in a boiling water bath for 10 minutes, then hybridized at 59°C for 1.5 hours. Following this, membranes were washed in solution B (965 mL distilled water, 25 mL 20× sodium citrate, 10 mL 10% sodium dodecyl sulfate) at 59°C for 15 minutes. The membranes were immersed in solution A [POD (peroxidase): solution A = 1:2000] for 30 minutes, then in a coloring solution [19 mL liquid C (900 mL distilled water, 100 mL 1M sodium citrate), 1 mL TMB, and 10 µL of 3% hydrogen peroxide] for 10 minutes. After rinsing with distilled water, a blue dot indicated positive detection. Positive and negative controls were included for each experiment (**Figure 1**).

### 2.5 Immunohistochemical staining

Consecutive 3µm sections were obtained from the same specimen for staining various immune cell markers. The sections were baked at 72°C for 30 minutes, labeled, and placed in an immunohistochemical staining machine (Benchmark XT, Ventana Medical Systems Inc., Tucson, USA). The primary antibody was added manually during the process. After completion, the sections were washed and bluing with hematoxylin for 5 seconds, then differentiated with hydrochloric acid, counterstained with ammonia water, dehydrated in graded ethanol, cleared in xylene, and coverslipped with neutral resin.

Information on the primary antibodies used is given below: CD3 (RabMAb, ZA-0503), CD4 (Murine monoclonal antibody, ZM-0418), CD8 (RabMAb, ZA-0508), CD20 (Murine monoclonal antibody, ZM-0039RUO), CD38 (Murine monoclonal antibody, ZM-0422), CD56 (Murine monoclonal antibody, ZM-0057), CD68 (Murine monoclonal antibody, ZM-0060), LCA (Murine monoclonal antibody, ZM-0183), PD-1 (Murine monoclonal antibody, ZM-0381), Ki-67 (Murine monoclonal antibody, ZM-0166), TTF-1 (Murine monoclonal antibody, ZM-0250), Napsin A (Murine monoclonal antibody, ZM-0473), CK (Murine monoclonal antibody, ZM-0069), CK7 (Murine monoclonal antibody, ZM-0071), P40 (Rabbit polyclonal antibody, ZM-0483), and CK5/6 (Murine monoclonal antibody, ZM-0313) were purchased from OriGene China;CTLA-4 (RabMAb, ab237712) and LAG-3 (RabMAb, ab237718)] were purchased from Abcam (Shanghai) Trading Co. Ltd; PD-L1 [SP263, (RabMAb, 08851620001)] was purchased from Ventana Medical Systems Inc. PBS was used as a negative control instead of primary antibody and known positive specimens were used as positive controls.

### 2.6 Interpretation of immunohistochemical staining results

The positive sites of CD3, CD56, PD-1, LAG-3 were located in the cytoplasm/membrane; the positive sites of CD4, CD8, CD20, CD38, LCA, PD-L1, CTLA-4 were located in the cell membrane; the positive sites of Napsin A, CK, CK7, CK5/6, CD68 are located in the cytoplasm; the positive sites of TTF-1, P40, Ki-67 were located in the cell nucleus. Upon microscopic examination of the slices, the appearance of specific yellow or brown granules in the respective sites was considered positive. The objective of TB tissue counting and observation was to focus on the granuloma area, whereas in the case of LC tissue, the tumor cell area was the primary area of interest.

The expression of LAC markers TTF-1, Napsin A, CK, CK7, P40 and CK5/6 in the adenocarcinoma region was observed microscopically, and the positive expression rate was counted. CD3, CD4, CD8, CD20, CD38, LCA, CD56, CD68, CTLA-4 counting criteria: randomly capture photos of 10 fields of view at 20× magnification in each stained slice and count the average number of positive cells. PD-L1, Ki-67, PD-1, LAG-3 lymphocyte counting criteria: observe the slide under the microscope and determine the percentage of positive cells. The assessment of LAG-3^+^ tumor cells involved interpreting the staining intensity, including “-”, “+”, “2+”, and “3+”, which represent 4 possible results. “-” indicated a negative result, meaning the cells were not stained. “+” indicated weak positivity, representing a low protein expression level. “2+” and “3+” indicated strong positivity, representing a high protein expression level. Two experienced pathologists jointly interpreted all slices to determine the results.

### 2.7 RNA extraction for sample sequencing

The tumor area samples were sectioned into eight consecutive 10 µm sections, which were placed in centrifuge tubes and dewaxed using xylene followed by anhydrous ethanol. Next, 200 µL of Digestion Buffer and 4 µL of protease were added and incubated at 50°C for 30 minutes, then at 80°C for 15 minutes. After that, 240 µL of Isolation Additive and 550 µL of absolute ethanol were mixed in. From this mixture, 700 µL was transferred to an RNeasy MinElute spin column in a 2 mL collection tube and centrifuged for 30 seconds. This process was repeated until the entire sample passed through the column. Then, 700 µL of Wash1, 500 µL of Wash2, and 500 µL of Wash3 were added sequentially, with centrifugation and waste disposal after each wash. After centrifuging for 2 minutes, the column was placed in a new tube, and 30-50 µL of Elution Solution was added to elute the RNA, which was then assessed for quality and quantity using an Agilent 2100 Bioanalyzer [Agilent Technologies (China) Ltd., Beijing, China]. RNA extraction was performed using the RecoverAll™ Total Nucleic Acid Isolation Kit for FFPE (Ingenics Life Technologies, Inc., Carlsbad, USA).

### 2.8 RNA sequencing

Library preparation was performed using the Optimal Dual-mode mRNA Library Prep Kit (BGI Genomics Co. Ltd, Shenzhen, China). RNA was denatured to open its secondary structure, allowing mRNA enrichment with oligo(dT) magnetic beads. The RNA was then fragmented, and first-strand cDNA was synthesized using random hexamer-primed reverse transcription, followed by second-strand cDNA synthesis. The double-strand cDNA underwent an end-repair reaction, with a single ‘A’ nucleotide added to the 3’ ends. Adaptors were ligated to the cDNA, which was then amplified via PCR and quality controlled. The products were denatured to create single-stranded libraries, then circularized to form single-stranded cyclized DNA, with linear DNA digested. The final circularized library was amplified using phi29 and rolling circle amplification (RCA) to produce DNA nanoballs (DNBs), each containing over 300 copies of the initial library molecule. These DNBs were sequenced on the DNBSEQ-T7 sequencer (BGI Genomics Co. Ltd, Shenzhen, China) using combinatorial probe anchor synthesis (cPAS).

### 2.9 Statistical analysis and sequencing data analysis

Images were processed using Photoshop (Adobe Systems Incorporated, San Jose, USA). Positive cells in the sections were counted using ImageJ (National Institutes of Health, Bethesda, USA). Data were compiled and analyzed using SPSS Statistics 20.0 (International Business Machines Corporation, Armonk, USA). Measurements following a normal distribution were reported as mean ± standard deviation (X̄*±* s), and comparison between groups was analyzed using one-way ANOVA and t-tests. Non-normally distributed data were presented as median [P50 (P25, P75)], and group comparison was analyzed using the Mann-Whitney U test. Comparisons of sample sizes (n) were performed using the chi-square or Fisher’s exact test. *P*<0.05 was used to represent a statistically significant difference.

The image data of the sequenced fragments obtained by the high-throughput sequencer were converted into sequence data (reads) through base calling using the CASAVA software (Illumina Inc., San Diego, USA). Rigorous quality control of raw data included removing reads with adapters, removing reads with N (N means that the base information cannot be determined), and removing low-quality reads. Paired-end clean reads were aligned using HISAT2 (24). Differential expression analysis between the two comparison groups was performed using DESeq2 software (Bioconductor, https://www.bioconductor.org/), and genes with an adjusted P-value of less than 0.05 were assigned as differentially expressed (Fisher’s exact test).

## 3 Results

### 3.1 Clinical and imaging features

In the TB group, there were 20 patients, which included 11 males and 9 females, with an average age of 52.3 years. The LAC group comprised 14 patients, consisting of 7 males and 7 females, with an average age of 60.6 years. The LAC-TB group also included 14 patients, made up of 6 males and 8 females, with an average age of 59.6 years. There were 7 cases of LAC combined with TB and 7 cases of LAC combined with peripheral lymph node TB in the lung. In comparing the gender, age, and smoking history among the three groups, the P-values were 0.784, 0.052, and 1.000, respectively, indicating no statistical differences. Likewise, when examining tumor diameter, tumor stage, cell differentiation, and metastasis between the LAC and LAC-TB groups, no statistically significant differences were found, with P-values of 1.000, 0.222, 1.000, and 0.234, respectively (**Table 1**).

**Table 1:** Comparison of clinical data and imaging features.

Classification	TB (n=20)	LAC (n=14)	LAC-TB (n=14)	*P*
TB type (MTB)	100% (n=20)	/	100% (n=14)	/
Acid-fast staining	100% (n=20)	/	100% (n=14)	/
Pathological Classification				
LAC	/	100% (n=14)	100% (n=14)	/
Age (year)	52.30 ± 11.18	60.64 ± 8.09	59.57 ± 12.06	0.052
*Gender*				0.784
Male	55% (n=11)	50% (n=7)	42.86% (n=6)	
Female	45% (n=9)	50% (n=7)	57.14% (n=8)	
*Smoking history*				1.000
Smoking	30% (n=6)	28.57% (n=4)	35.71% (n=5)	
Non-smoking	70% (n=14)	71.43% (n=10)	64.29% (n=9)	
*Site of TB lesion*				0.001
Pulmonary	100% (n=20)	/	50% (n=7)	
Peripulmonary lymph nodes	0 (n=0)	/	50% (n=7)	
*Tumor Diameter*				1.000
≥5cm	/	7.14% (n=1)	14.29% (n=2)	
<5cm	/	92.86% (n=13)	85.71% (n=12)	
*Tumor Stage*				0.222
I-II	/	100% (n=14)	78.57% (n=11)	
III-IV	/	0 (n=0)	21.43% (n=3)	
*Cell Differentiation*				1.000
Well-differentiated	/	42.86% (n=6)	50% (n=7)	
Moderately differentiated	/	50% (n=7)	42.86% (n=6)	
Poorly differentiated	/	7.14% (n=1)	7.14% (n=1)	
*Metastasis*				0.234
Hematogenous metastasis	/	0 (n=0)	0 (n=0)	
Lymph node metastasis	/	0 (n=0)	21.43% (n=3)	
CT (Pulmonary)				
Ground-Glass opacity shadows	10% (n=2)	42.86% (n=6)	35.71% (n=5)	0.059
Irregular nodular shadows	65% (n=13)	35.71% (n=5)	42.86% (n=6)	0.199
Density foci of soft tissue masses	25% (n=5)	21.43% (n=3)	21.43% (n=3)	1.000
Burr	10% (n=2) ^*^	28.57% (n=4)	50% (n=7)	0.031
Lobular	20% (n=4)	42.86% (n=6)	42.86% (n=6)	0.250

Asterisks represent statistically significant differences between the TB and LAC-TB groups.

**Figure 2:**
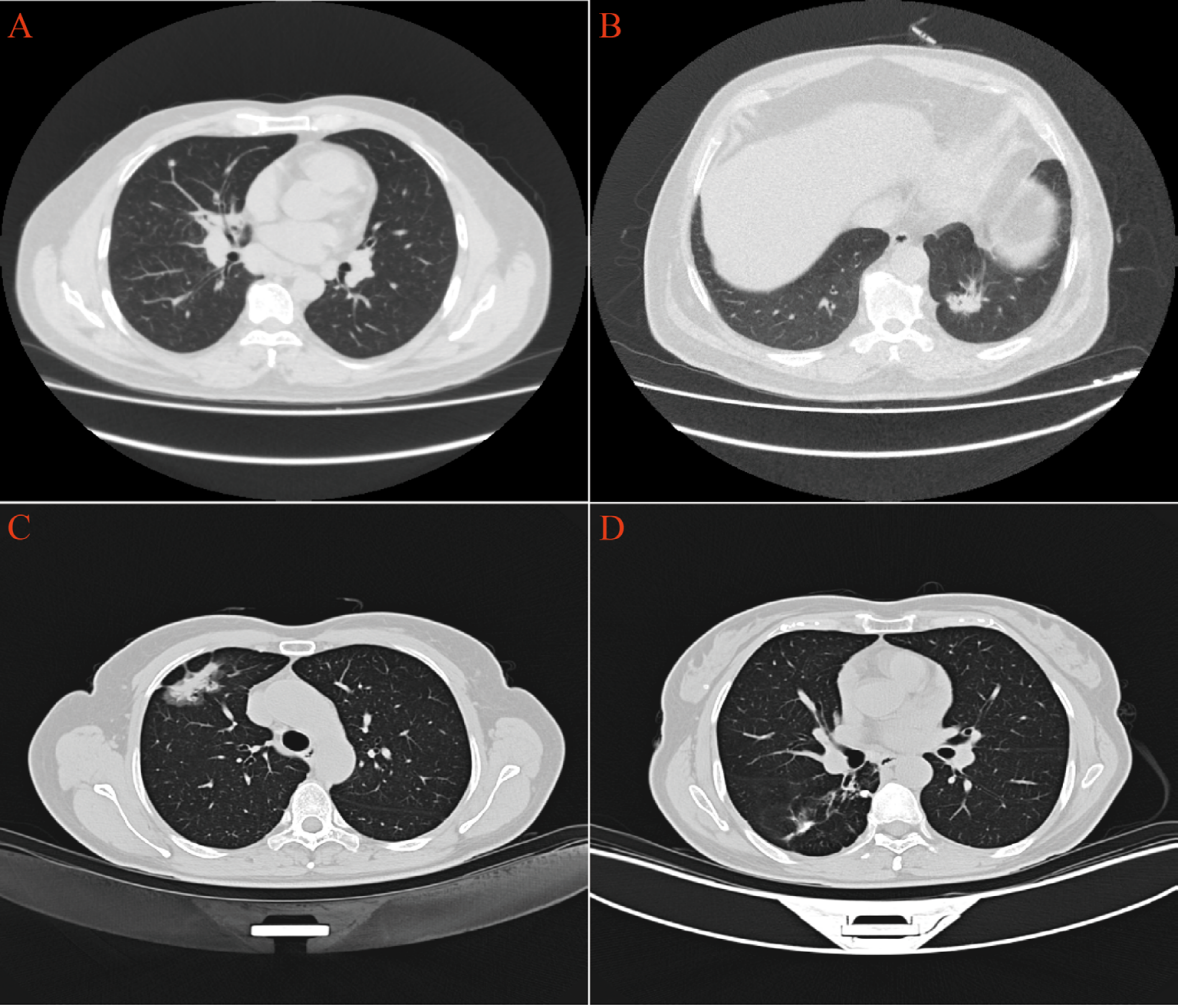
CT imaging revealed distinct pulmonary manifestations across the three study groups. **(A)** TB group exhibited scattered small nodules throughout the lung parenchyma. **(B)** LAC group presented with irregular nodular shadows in the lungs. **(C)** In the LAC-TB group, LAC lesions demonstrated both nodular and ground-glass shadows. **(D)** In the LAC-TB group, TB lesions displayed scattered nodular calcifications.

CT scans have shown that some LAC lesions can present as ground-glass opacity (GGO) shadows and irregular nodular shadows, often exhibiting lobular characteristics. In certain cases, lungs affected by LAC-TB displayed burr-like features in addition to typical LAC characteristics. However, these features did not significantly differ from those in the LAC group and were notably different from the TB group, with a P-value of 0.031(**Figure 2**, **Table 1**). The distinction between LAC and TB on imaging is sometimes difficult to distinguish. Pathological examination is necessary to diagnose LC combined with TB or lymph node TB. Detecting TB foci on preoperative imaging is essentially difficult.

### 3.2 MTB infection increases Ki-67 proliferation index in LAC

**Figure 3:**
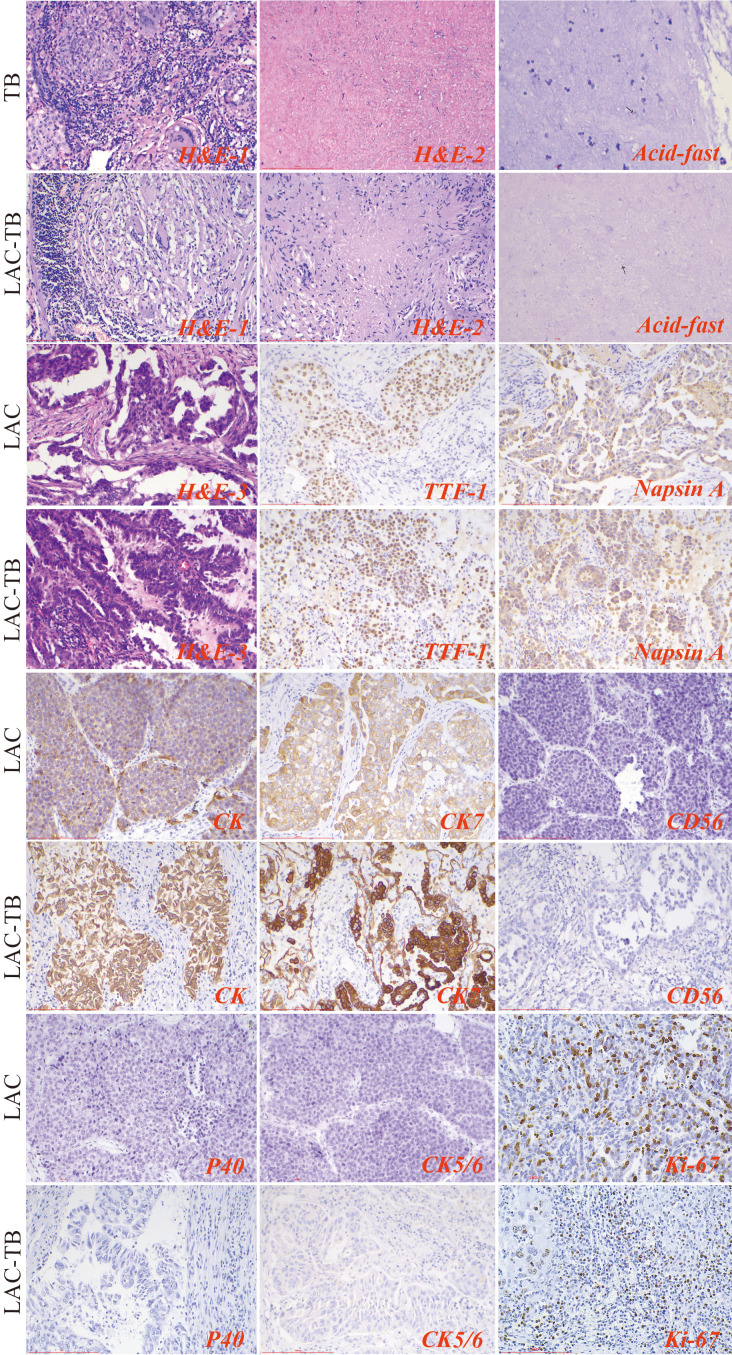
Results of H&E staining, acid-fast staining, and immunohistochemical staining in the three groups. The TB lesions in both the TB and LAC-TB groups exhibited typical pathological changes of granulomatous inflammation (H&E-1), accompanied by caseous necrosis (H&E-2), and MTB was identified through acid-fast staining (acid-fast; black arrows indicate MTB). A significant number of diverse glands, mainly of the follicular type, were observed in the tumor lesion areas in both the LAC and LAC-TB groups (H&E-3). Immunohistochemical analysis revealed positive staining for LAC-specific markers (TTF-1 and Napsin A), as well as CK and CK7, in both the LAC and LAC-TB groups. These groups were negative for CD56, P40, and CK5/6. Ki-67 expression, a marker of cellular proliferation, was also assessed. H&E staining (200×), Scale bar: 200 µm; Acid-fast staining (630×), Scale bar: 100 µm; Immunohistochemical staining (200×), Scale bar: 200 µm.

Histopathological examination of the TB group exhibited chronic granulomatous inflammation, caseous necrosis, and MTB confirmation via molecular and acid-fast staining. The LAC group revealed predominantly follicular-type glandular structures with varying lymphocytic infiltration in the tumor stroma. The LAC-TB group showed a glandular structure similar to that of the LAC group, which was characterized by lymphocytic infiltration. Additionally, adjacent TB lesions, including granulomas and caseous necrosis, were found in the peritumoral region or the peripulmonary lymph nodes. MTB infection was also confirmed in this group. (**Figure 3**)

The LAC and LAC-TB groups expressed adenocarcinoma markers TTF-1 and Napsin A, along with CK and CK7, but lacked CD56, P40, and CK5/6 expression (**Figure 3**, **Supplementary Table 1**). Immunohistochemical analysis revealed a higher proportion of cells with a Ki-67 proliferation index ≥10% in the LAC-TB group compared to the LAC group (*p*=0.021), indicating increased cellular proliferation in the LAC-TB group (**Supplementary Table 2**).

### 3.3 MTB infection increases the degrees of malignancy in LAC

**Figure 4:**
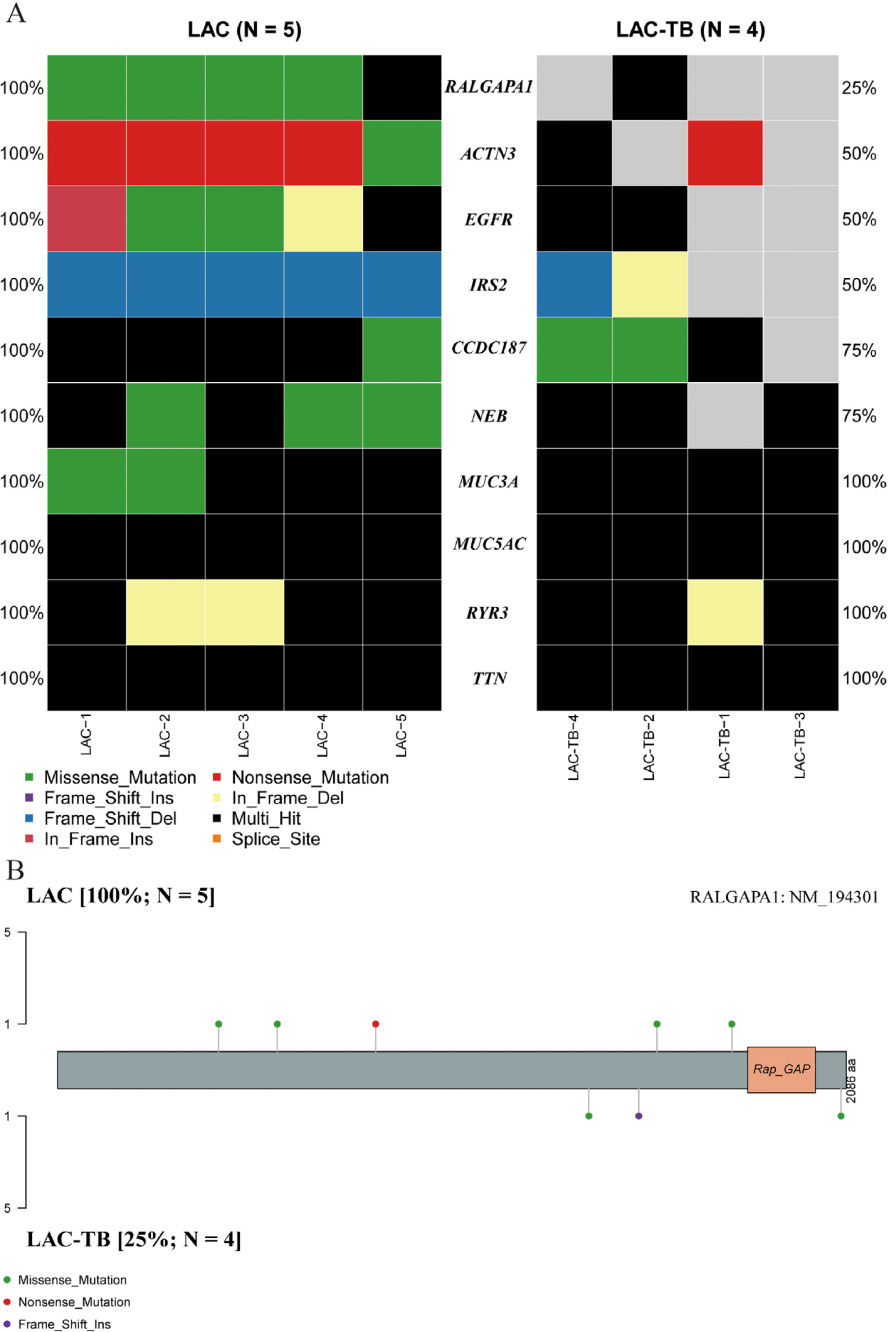
Genetic divergence between the LAC and LAC-TB groups. **(A)** Comparative analysis of mutation frequencies in ten genes revealed differences between the LAC-TB and LAC groups, specifically in *RALGAPA1*, *ACTN3*, *EGFR*, *IRS2*, *CCDC187*, and *NEB*. The RALGAPA1 gene exhibited a significantly higher mutation frequency in the LAC group (100%) compared to the LAC-TB group (25%). **(B)** A lollipop plot depicting the protein domain architecture of RALGAPA1 highlighted the specific amino acid residues affected by mutations, along with the nature of these mutations. The mutation profiles of the two groups differed. The LAC group exhibited missense and nonsense mutations, while the LAC-TB group showed missense and frame-shift insertion mutations.

A comprehensive genomic analysis of LAC and LAC-TB samples revealed a significant difference in the frequency of *RALGAPA1* mutations (*p*<0.05). All LAC samples exhibited *RALGAPA1* mutations, while only one LAC-TB sample did (**Figure 4A**, **Supplementary Figure 1**). Both groups contained missense mutations; however, frameshift insertions were exclusive to the LAC-TB group, and nonsense mutations were found only in the LAC group (**Figure 4B**).

**Figure 5:**
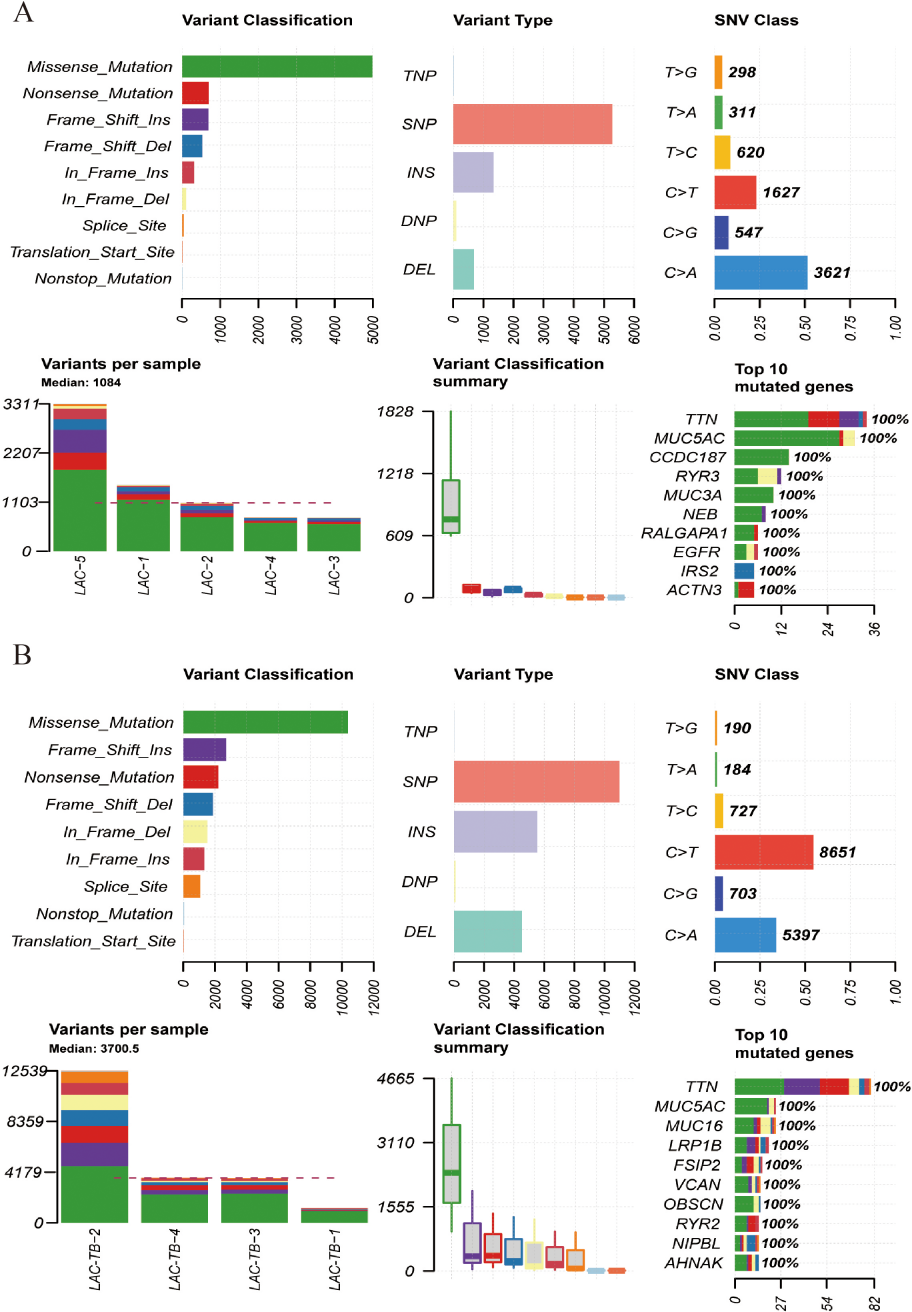
Mutational landscape of the LAC and LAC-TB groups. **(A)** Overview of the mutational profile in the LAC group, including variant classification, variant type, single nucleotide variant (SNV) class, variants per sample, and the ten most frequently mutated genes. Missense mutations constituted the most prevalent variant classification, with SNP being the predominant variant type. C>A and C>T transitions were the more common categories of SNVs observed. The median number of variants per sample was 1084. The ten most frequently mutated genes were *TTN, MUC5AC, CCDC187, RYR3, MUC3A, NEB, RALGAPA1, EGFR, IRS2*, and *ACTN3*. **(B)** Overview of the mutational profile in the LAC-TB group, including variant classification, variant type, SNV class, variants per sample, and the ten most frequently mutated genes. Missense mutations were the most common type of variant classification, with SNP being the predominant variant type. C>T and C>A transitions were the most commonly observed types of SNVs. On average, there were 3700.5 variants per sample. The ten genes with the highest mutation frequency were *TTN, MUC5AC, MUC16, LRP1B, FSIP2, VCAN, OBSCN, RYR2, NIPBL*, and *AHNAK*.

Additionally, both groups showed a higher ratio of C>A and C>T base substitutions (**Supplementary Figure 2**), with missense mutations being the most prevalent variant classification and single nucleotide polymorphism (SNP) as the most common type of variant (**Figure 5**). In the LAC-TB group, both missense mutations and SNP were more prevalent compared to the LAC group. Notably, the LAC-TB group displayed a significantly higher median mutation count (3700.5) than the LAC group (**Figure 5**).

**Figure 6:**
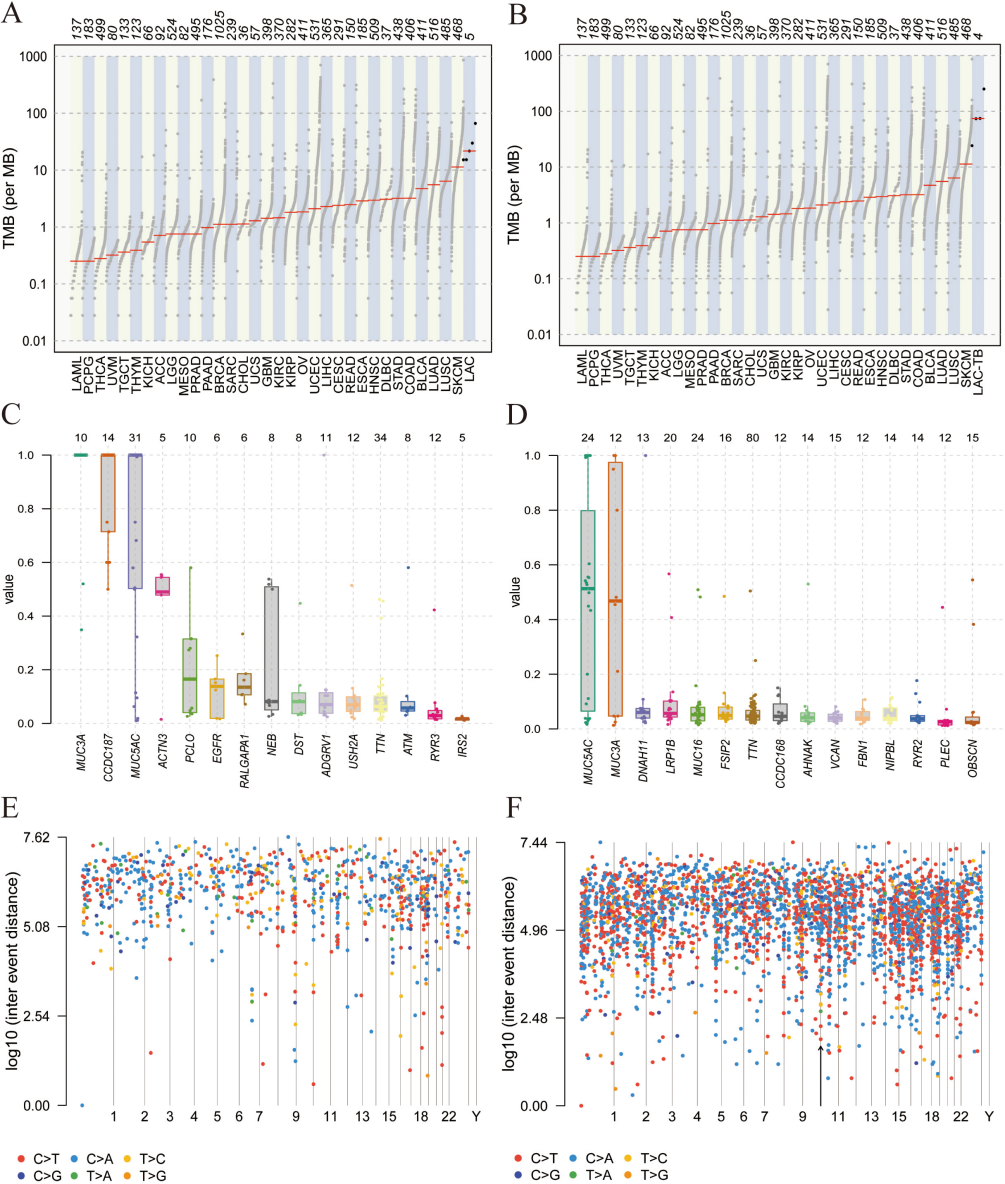
Mutational analysis of the LAC and LAC-TB groups. **(A)** A comparison of TMB in the LAC group with other tumor types demonstrated a higher median TMB in LAC. **(B)** TMB analysis showed that the median TMB in the LAC-TB group was approximately 100 greater than in other tumor types. **(C)**VAF distribution in the LAC group showed a frequency approaching 50% only for the ACTN3 gene. **(D)** VAF distribution in the LAC-TB group reveals frequencies nearing 50% for both *MUC5AC* and *MUC3A*. **(E)** Distribution of mutation spacing across chromosomes in the LAC group. **(F)** Chromosomal distribution of mutation spacing in the LAC-TB group, with regions of ultra-high mutational density indicated by black arrows.

Tumor Mutation Burden (TMB) refers to the number and frequency of mutations in tumor cells, indicating genomic instability and tumor evolution. Our study found that TMB was higher in the LAC-TB group than in the LAC group (**Figures 6A, B**). **Figures 6C, D** show the Variant Allele Frequency (VAF) distribution for 15 genes in both groups, while **Figures 6E, F** illustrate the hyper-mutated genomic regions for the LAC and LAC-TB groups.

### 3.4 MTB infection reduces the number of infiltrating T cells and CD4^+^ T cells in LAC

**Figure 7:**
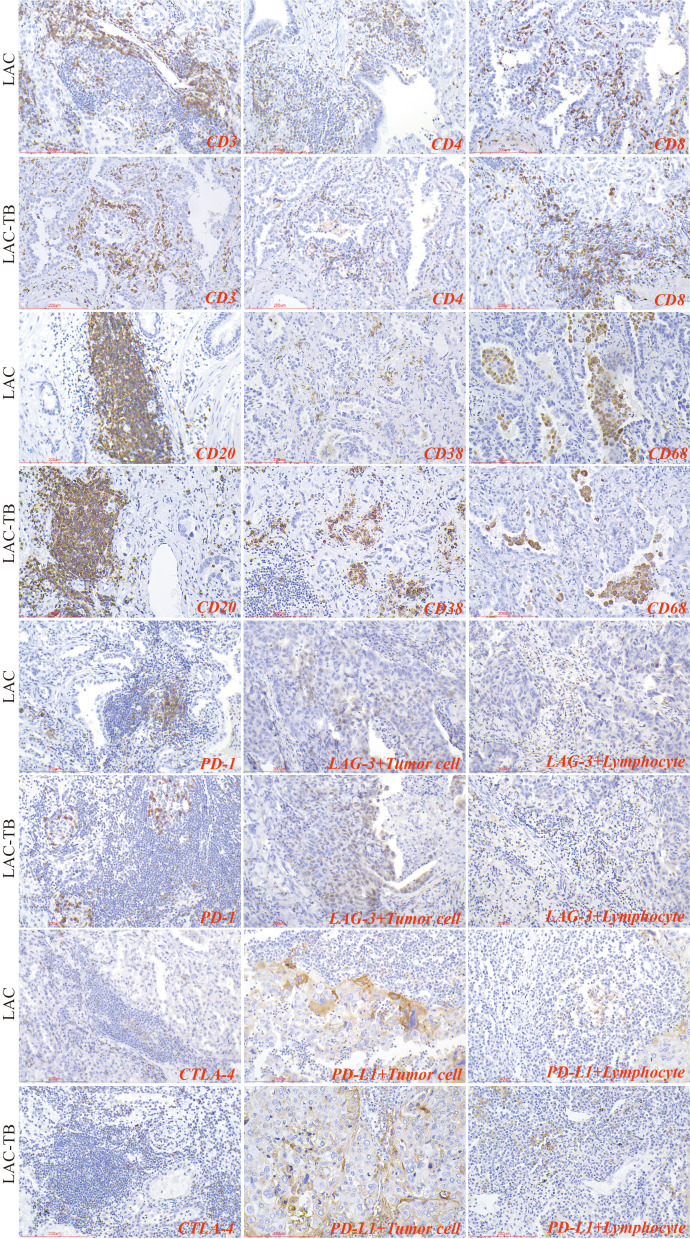
Immunohistochemical characterization of immune cell markers and immune checkpoints within the TME of the LAC and LAC-TB groups. CD3, CD4, and CD8 are markers used to identify T cells, while CD20 is a marker for B cells, CD38 indicates plasma cells, and CD68 is associated with macrophage cells. Additionally, the expression of immune checkpoint molecules was evaluated, including PD-1 on lymphocytes, CTLA-4 on T cells, and PD-L1 and LAG-3 on both lymphocytes and tumor cells. Immunohistochemical staining (200×), Scale bar: 200 µm.

Lymphocytes were found in a focal distribution within LAC, primarily T and B cells, with macrophages present and natural killer (NK) cells being the least common. In the LAC-TB group, CD3^+^ T cells decreased by 17.87% and CD4^+^ T cells by 28.16% compared to the LAC group. The number of plasma cells was significantly higher in the LAC-TB group (*p*<0.001), though the significance of this difference is unclear. Other cell types, including LCA^+^ lymphocytes, CD8^+^ T cells, CD20^+^ B cells, NK cells, and macrophages, showed no significant differences between the two groups, with P-values of 0.648, 0.960, 0.773, 0.851, and 0.409, respectively. These results suggest that MTB infection suppresses anti-tumor immunity in LAC patients, mainly affecting CD3^+^ and CD4^+^ T cells (**Figure 7**, **Table 2**). Immunohistochemical staining of CD56 and LCA in the tumor regions is shown in **Supplementary Figure 3**

**Table 2:** Infiltration of various types of immune cells and expression of immune checkpoint molecules in the LAC and LAC-TB groups.

Type	LAC [(n), (%)]	LAC-TB [(n), (%)]	*P*
Immune cell type			
CD3^+^ T cell	741.56 ± 484.86	609.01 ± 399.04	0.013
CD4^+^ T cell	581.26 ± 411.05	417.58 ± 312.47	<0.001
CD8^+^ T cell	400.24 ± 230.18	401.51 ± 189.02	0.960
CD20^+^ B cell	665.04 ± 651.53	642.56 ± 651.55	0.773
CD38^+^ plasma cell	268.51 ± 216.24	484.43 ± 424.60	<0.001
CD56^+^ NK cell	0.43 ± 0.98	0.36 ± 1.20	0.851
CD68^+^ macrophage cell	335.63 ± 245.24	315.17 ± 159.38	0.409
LCA^+^ lymphocyte cell	837.44 ± 581.01	805.10 ± 603.61	0.648
Checkpoint molecules type			
PD-L1^+^ tumor cell	1.00 (1.00, 1.00)	5.00 (1.75, 40.00)	0.001
CTLA-4^+^ T cell	136.00 (67.50, 299.25)	124.50 (56.25, 242.25)	0.435
PD-1^+^ lymphocyte cell	5.00 (4.25, 10.00)	7.50 (3.00, 16.25)	0.357
PD-L1^+^ lymphocyte cell	2.00 (1.00, 5.00)	5.00 (1.00, 10.00)	0.150
LAG-3^+^ lymphocyte cell	10.00 (8.75, 16.25)	5.00 (1.00, 11.25)	0.033

### 3.5 MTB infection promotes PD-L1 expression in LAC cells

In summary, PD-1 and CTLA-4 were primarily expressed in lymphocytes, whereas PD-L1 and LAG-3 were present in both tumor and lymphocyte cells. The proportion of PD-L1^+^ LAC cells in the LAC-TB group was four times higher than in the LAC group (*p*=0.001). Additionally, LAG-3^+^ lymphocytes decreased by 50% in the LAC-TB group compared to the LAC group (*p*=0.033). No significant differences were found for PD-L1^+^ lymphocytes, CTLA-4^+^ T cells, PD-1^+^ lymphocytes, and LAG-3^+^ LAC cells, with P-values of 0.150, 0.435, 0.357, and 0.272, respectively (**Figure 7**, **Table 2**, **Supplementary Table 3**).

### 3.6 Strong positive expression of PD-L1 in areas of TB lesions

**Figure 8:**
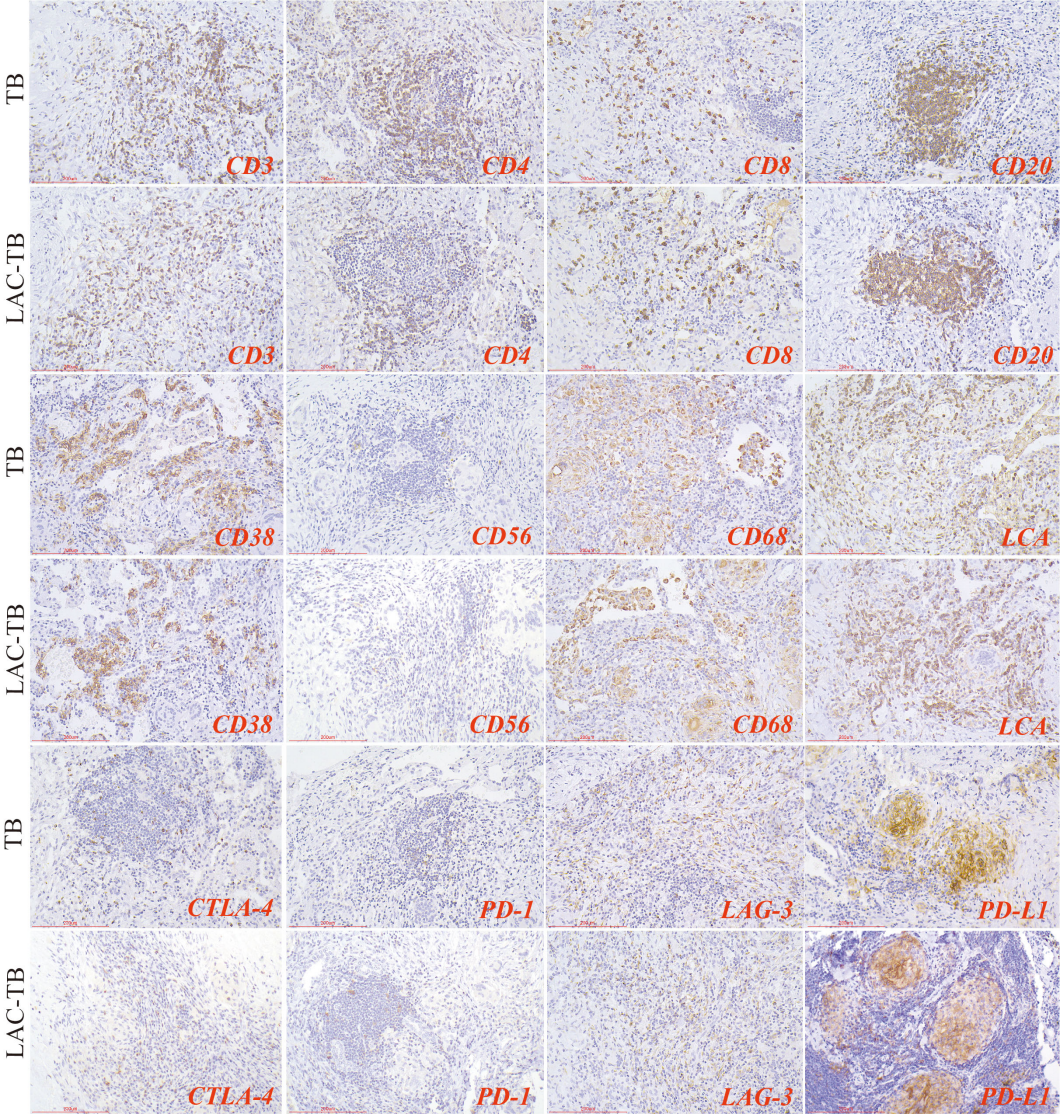
Immunohistochemical staining for immune cell markers and immune checkpoint molecules in TB and LAC-TB granuloma regions. CD3, CD4, and CD8 are markers used to identify T cells, while CD20 serves as a marker for B cells and CD38 identifies plasma cells. CD56 is used for natural killer (NK) cells, CD68 marks macrophages, and LCA labels lymphocytes. Additionally, the expression of immune checkpoint molecules was evaluated, including PD-1 on lymphocytes, CTLA-4 on T cells, and PD-L1 and LAG-3 on lymphocytes. Immunohistochemical staining (200×), Scale bar: 200 µm.

We analyzed immune cells in TB lesions of the LAC-TB and TB groups and found no significant differences (**Table 3**). However, immune checkpoint molecules such as CTLA-4, PD-1, LAG-3, and PD-L1 were present in the granuloma region of both groups (**Figure 8**). PD-L1 expression was significantly higher in the LAC-TB group compared to the TB group, with a P-value of 0.021 (**Table 3**).

**Table 3:** Infiltration of various types of immune cells and expression of immune checkpoint molecules in areas of TB lesions of the TB and LAC-TB groups.

Type	TB [ (n), (%)]	LAC-TB [ (n), (%)]	*P*
Immune cell type			
CD3^+^ T cell	982.22 ± 437.63	927.60 ± 660.14	0.360
CD4^+^ T cell	764.27 ± 438.57	698.00 ± 327.88	0.131
CD8^+^ T cell	624.01 ± 329.78	658.60 ± 108.82	0.233
CD20^+^ B cell	1052.96 ± 721.15	946.40 ± 461.72	0.124
CD38^+^ plasma cell	692.63 ± 452.59	730.00 ± 172.93	0.353
CD56^+^ NK cell	2.00 + 2.50	1.00 ± 1.00	0.167
CD68^+^ macrophage cell	798.35 ± 393.46	838.00 ± 250.45	0.293
LCA^+^ lymphocyte cell	1258.27 ± 546.96	1139.40 ± 556.46	0.051
Checkpoint molecules type			
PD-L1^+^ lymphocyte cell	70.00 (52.50,80.00)	90.00 (73.75,90.00)	0.021
CTLA-4^+^ T cell	387.63 ± 235.25	436.40 ± 285.26	0.086
PD-1^+^ lymphocyte cell	10.00 (5.00,15.00)	5.00 (1.00,13.75)	0.091
LAG-3^+^ lymphocyte cell	17.50 (15.00,25.00)	12.50 (5.00,62.50)	0.578

## 4 Discussion

Diagnosing LC-TB is challenging due to their similar clinical and imaging characteristics. Pathology is vital in distinguishing between the two. In LC, pathology determines tumor type, grade, and stage, guiding treatment plans. For TB, histological examinations confirm diagnoses and reveal pathological changes in lung tissues. The coexistence of LC and TB is common, making accurate pathology essential for effective diagnosis and treatment. This understanding helps clarify the characteristics of LC-TB and supports the development of comprehensive treatment strategies.

Some studies show that the relationship between TB and LC is not significant (25, 26), while other studies find that the risk of developing LC is increased by 1.57-83.7 times in patients with TB compared to those without TB infection (27, 28). Current research indicates that MTB infection may impact cancer development and progression through multiple mechanisms. The chronic inflammation associated with TB can foster a pro-tumor environment, encouraging angiogenesis, cell proliferation, and immune evasion (29, 30). Furthermore, the immunosuppressive effects of TB can impair the host’s ability to effectively eliminate tumor cells (31). Studies have shown an association between MTB infection and an increased risk of various cancers, including LC (32, 33). Nonetheless, the exact mechanisms behind this association and their effects on the tumor’s biological characteristics remain poorly understood.

This study provides strong evidence of a complex relationship between LAC and TB co-infection, which affects the malignancy and immunogenicity of adenocarcinoma. Our study suggests that MTB infection may not only directly influence the TME but also create a microenvironment promoting immune evasion by the tumor cells through PD-L1-mediated suppression of anti-tumor immunity. This finding aligns with previous research demonstrating the immunosuppressive effects of MTB infection (34, 35). The potential for TB-induced chronic inflammation to promote tumorigenesis has been previously explored (10, 36), and our findings lend further support to this hypothesis.

The notable difference in *RALGAPA1* mutation frequency between the LAC-TB and LAC groups suggests further investigation into its role in LAC pathogenesis. The unexpectedly lower frequency of mutations in the LAC-TB group indicates a complex interaction between MTB infection and tumorigenesis. Although the exact function of *RALGAPA1* in LAC is not yet fully understood, current literature indicates its role in various cellular processes, such as cell growth, differentiation, and apoptosis (37, 38). Thus, the lower mutation rate in the LAC-TB group may suggest either selective pressure from the MTB infection on *RALGAPA1*-mutated cells or an alternative tumor development pathway due to co-infection.

Several hypotheses can explain this intriguing finding. First, the chronic inflammation caused by MTB infection may alter the selective pressures within the TME. This change could favor the growth of LAC clones with a different mutational profile, which includes a lower frequency of *RALGAPA1* mutations. Second, immune responses triggered by MTB, such as the recruitment of immune cells and the release of inflammatory cytokines, may indirectly influence the mutational landscape of tumor cells. This influence could either suppress or promote specific mutations, depending on the complex interactions between the immune system and the tumor. Third, the observed differences in *RALGAPA1* mutation frequency might reflect distinct molecular pathways of LAC development in the presence and absence of MTB infection. *RALGAPA1* mutations may be critical for LAC progression without MTB, while alternative oncogenic pathways could be activated during co-infection, reducing dependence on *RALGAPA1* mutations for tumor development.

The lower mutation rate of *RALGAPA1* in the LAC-TB group, combined with a higher overall mutation burden and an increased Ki-67 proliferation index, suggests that MTB infection may be associated with a more aggressive form of LAC that has distinct molecular characteristics. Further research is needed to understand the specific mechanisms behind the observed differences in *RALGAPA1* mutations and their implications for tumor progression and treatment strategies in LAC-TB patients. Functional studies examining the role of *RALGAPA1* in the context of MTB infection are essential to clarify the biological significance of these findings.

MTB infection can induce the production of reactive oxygen species (ROS), leading to oxidative stress and DNA damage, which may result in the accumulation of gene mutations, thus driving the development of LC. Compared to LC, TB patients show defects in cell division and DNA repair functions, leading to a higher rate of DNA damage accompanied by higher levels of apoptosis, necrosis, and impaired cell division (39). This explains the considerable discrepancies in mutation data observed between the LAC-TB and LAC groups in the course of this study. MTB infection can alter DNA methylation, histone modification, and microRNA expression, affecting the expression of tumor suppressor genes and oncogenes.

The observed increase in PD-L1 expression and elevated TMB in patients with LAC-TB have important clinical implications for treatment strategies. High PD-L1 expression, which tumors use as a key mechanism to evade the immune system, indicates a potential role for ICIs, such as anti-PD-1 or anti-PD-L1 antibodies, in treating LAC-TB. These therapies can help restore anti-tumor immunity by blocking the PD-1/PD-L1 interaction, thus allowing T-cells to become more active and effective in attacking tumors (40). Additionally, the high TMB identified in this study may suggest a better response to ICIs therapy. In a broad sense, Tumor mutational burden (TMB) refers to the number of somatic mutations per megabase of the genome sequence, which varies depending on the type of tumor (41). Increasing evidence suggests that TMB may serve as a predictive biomarker for tumor response to ICIs in several types of cancers (42, 43), particularly showing robust initial responses to ICIs in NSCLC (44). Generally, cancers with high TMB exhibit a greater number of mutations, which may result in the generation of more neoantigens, triggering an immune response and thus possessing stronger potential for immunotherapy or targeted therapy. In contrast, tumors with low TMB demonstrate a reduced response to these types of treatment. Recent studies have confirmed the link between high TMB and better outcomes in various cancers treated with ICIs (45, 46). The infection of MTB increases the TMB of LAC. Theoretically, patients with LAC-TB should exhibit a stronger response to immunotherapy. ICIs function by stimulating the immune system to recognize and target cancer cells, thereby exerting therapeutic effects on tumors. However, in this study, patients in the LAC-TB group showed a significant decrease in CD3^+^ T cells and CD4^+^ T cells in the TME, indicating that the immune response is suppressed, which may weaken the effectiveness of immunotherapy.

Research has found that immunotherapy is relatively safe for LC patients with latent TB who have undergone previous treatment, and the efficacy of immunotherapy in this specific population is comparable to LC patients without TB infection (21). However, the presence of concurrent TB infection adds complexity and requires careful consideration when implementing immunotherapy. A significant concern regarding LAC-TB is the risk of reactivation of latent TB infection (47). The median progression-free survival (PFS) in tumor immunotherapy of LC patients with active TB infection is shortened by more than half (21). Multiple case reports and studies have documented TB reactivation following ICIs therapy, emphasizing the importance of vigilant monitoring and proactive management (48, 49).

Careful monitoring for signs and symptoms of TB reactivation is essential for patients with LAC-TB who are receiving immunotherapy. Signs to watch for include fever, cough, weight loss, and swollen lymph nodes (lymphadenopathy). It is recommended to perform baseline screening for latent TB infection using interferon-gamma release assays (IGRAs) or tuberculin skin tests (TSTs) (47). For patients with latent TB, it is advisable to consider prophylactic anti-TB therapy before starting ICIs treatment to reduce the risk of reactivation. The ideal duration and regimen for this prophylactic therapy are still under investigation. Additional research is necessary to refine treatment strategies for LAC-TB, ensuring a careful balance between the potential benefits of immunotherapy and the risks of TB reactivation. Prospective clinical trials that evaluate the efficacy and safety of ICIs in LAC-TB patients, alongside optimized strategies for TB prophylaxis and management, are crucial for improving care for this complex patient population.

PD-L1 is an immune checkpoint protein expressed on tumor cells and tumor-infiltrating immune cells, mediating immune suppression against cancer by binding with its receptor PD-1. Trials of inhibitors targeting PD-1 and PD-L1 rely on the expression of PD-L1 as a biomarker to predict clinical benefits (50). After MTB infection, the expression of PD-L1 on antigen-presenting cells may bind with PD-1 on lymphocytes and activate downstream pathways, leading to lymphocyte immune exhaustion (51). It has been reported that the activation of the PD-1/PD-L1 pathway is involved in T cell dysfunction during tumor development, but its role in the pathogenesis of MTB infection remains controversial (51). The increased expression of PD-L1 in TB patients is associated with higher bacterial burden and its expression is related to the progression and treatment of TB (52). Studies have found significant expression of PD-L1 in granulomatous prostatitis, but not in the tumor parenchyma and stroma of prostate adenocarcinoma (53). However, in this study, PD-L1 is highly expressed in tumor cells and tuberculous granulomatous areas in the LAC-TB group. This may be due to the transmission of PD-L1 signal by MTB infection through exosomes or genomic integration, leading to high expression of PD-L1 in LC cells.

Exosomes are biologically active lipid bilayer nanovesicles released by almost all types of normal cells and malignant cells (54). TEXs can transmit signals between tumor cells and immune cells (55). Various immune checkpoint proteins exist in exosomes, especially those derived from tumors, such as PD-L1 (56). Exosomal PD-L1 can inhibit the effector function of T cells, induce systemic immune suppression, and transfer functional PD-L1 to the TME (57). We are currently unable to verify this mechanism, and further collection and purification of exosomes from the plasma of more LAC-TB patients are needed to detect exosomal PD-L1. In addition, since MTB is an intracellular pathogen, it may play a positive role in cell transformation (58). Studies have shown the presence of MTB IS6110 transposon in the tumor tissue genome of LAC patients, with the existence of IS6110 found in the nuclei of tumor cells (59). After MTB infection, integrating its DNA into the normal cell genome may provide a new mechanism for the occurrence of LC through disrupting oncogenes.

In light of the results of this study, MTB infection transfers PD-L1 to normal cells, causing partial expression of PD-L1 in normal cells to evade immune clearance by the immune system, leading to continued proliferation and division, and ultimately triggering the occurrence of LC. This may explain the high expression of PD-L1 in tumor cells and TB granulomatous areas in the LAC-TB group.

However, a major limitation of this study is the small sample size, especially within the LAC-TB group (n=14), which may not reflect the broader population. While significant differences between the LAC-TB and LAC groups suggest strong effects, the small size diminishes the ability to detect smaller, yet potentially biologically relevant, differences. Furthermore, a power calculation was not performed beforehand to establish the required sample size for specific variables. Thus, larger studies are needed to validate these findings.

Several factors, however, support the use of this sample size for the current investigation. First, the study focuses on identifying significant differences between two groups, which often requires a smaller sample size for statistical significance. The large effect sizes observed for key variables, like the Ki-67 proliferation index and distinct mutational profiles, affirm the reliability of our findings despite the small sample. Second, obtaining LAC-TB patient samples is challenging due to the rarity of this co-infection and difficulties in accessing suitable tissue specimens. This sample size represents a substantial effort for this understudied condition. Finally, significant differences in parameters such as the Ki-67 index, mutation load, and *RALGAPA1* expression provide strong evidence supporting our conclusions. Thus, while modest, the sample size is adequate to reveal clear differences between the two groups.

Future research involving larger sample sizes is needed to validate these findings and investigate the underlying mechanisms in more detail. Additionally, using a prospective study design would enable more accurate power calculations and a stronger assessment of the relationship between MTB infection and LAC malignancy. Despite the limitations due to the small sample size, this study offers valuable preliminary data that highlights the significant impact of MTB infection on the biological characteristics of LAC.

## 5 Conclusions

*Mycobacterium tuberculosis* infection increases the expression level of Ki-67 in lung adenocarcinoma and enhances the malignancy of lung adenocarcinoma at tissue and molecular levels. The mechanism may involve the delivery of PD-L1 signals from granulomatous areas, inhibiting T cells and CD4^+^ T cells, and weakening anti-tumor immunity.

## Data Availability

The sequencing data have been uploaded to public databases, and the rest of the original contributions presented in the study are included in the article/**Supplementary Material**. For database links, please contact the corresponding author.
