# Supplementary material for: Mycobacterium tuberculosis infection may increase the degrees of malignancy in lung adenocarcinoma
Source: Front Immunol. 2025 Feb 21;16:1537520. doi: 10.3389/fimmu.2025.1537520 (PMC11885956; doi:10.3389/fimmu.2025.1537520)
Supplement: Supplementary file 1 [file DataSheet1.docx]

Supplementary Material

# Supplementary Figures and Tables

## Supplementary Figures


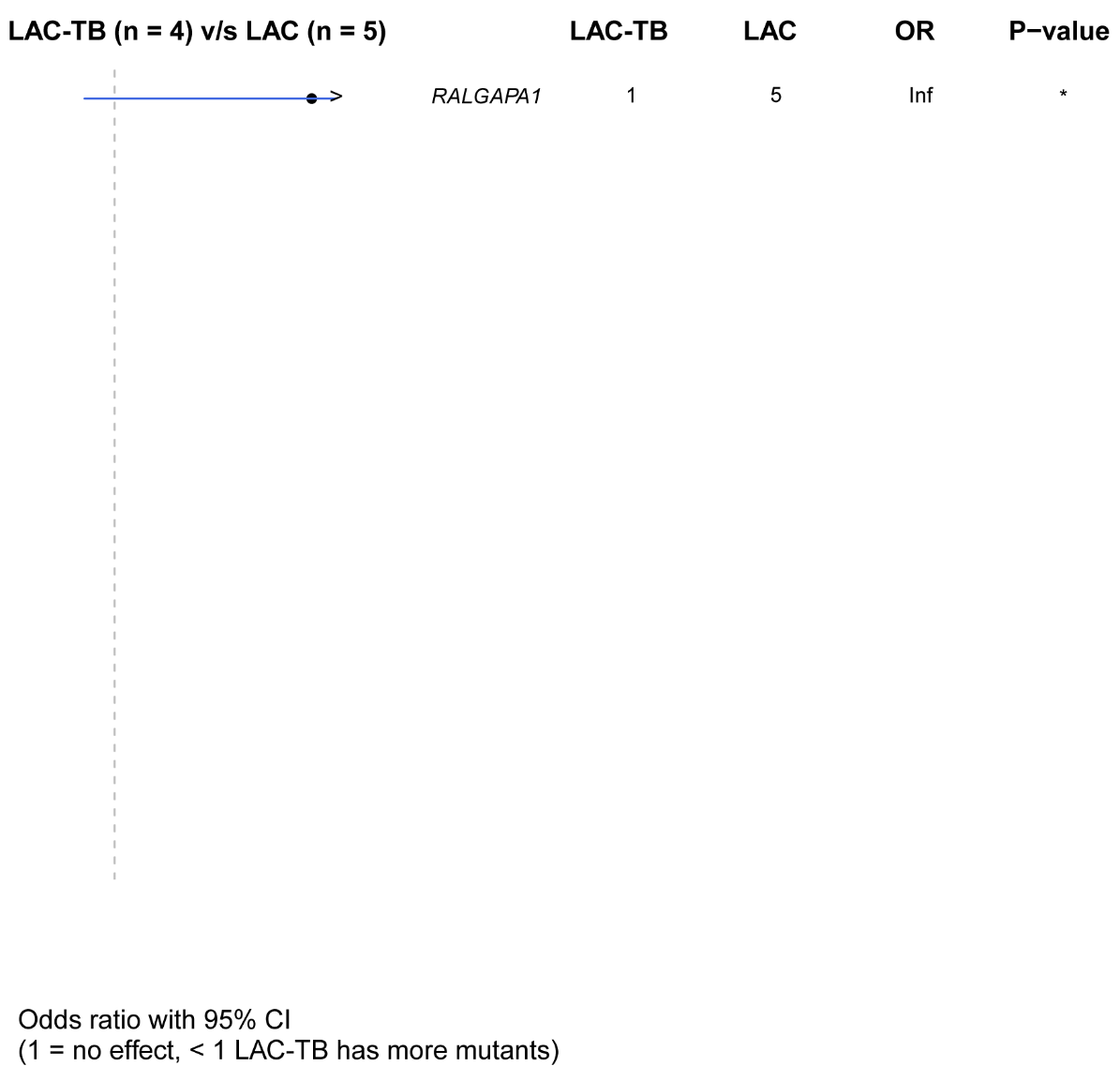


**Supplementary Figure 1.** A differentially mutated gene between the LAC-TB and LAC groups was shown in a forest plot. Bars indicated 95% confidence intervals for the Odds ratio. The table on the right includes the number of samples in LAC-TB and LAC for each of the mutations in the gene highlighted by the P-value. P-value indicated threshold significance: (*) *P* < 0.05; Fisher's exact test.


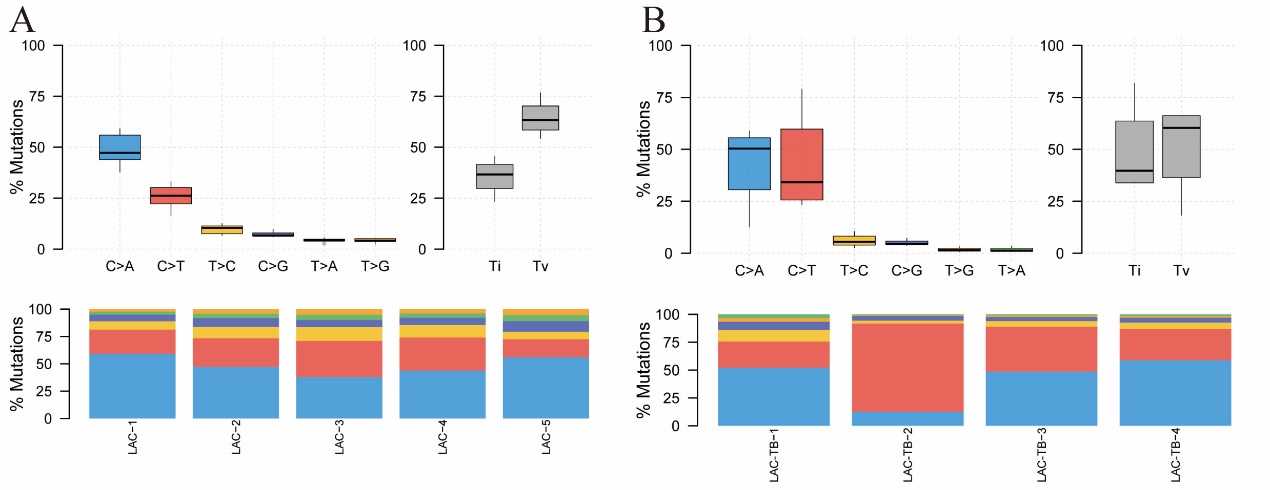


**Supplementary Figure 2.** The mutation data of the LAC and LAC-TB samples. **(A)** The transition and transversion plot (Ti/Tv) showed the distribution of single nucleotide variation (SNV) in LAC with a stacked bar plot at the bottom displaying the mutational spectrum distribution of each sample. **(B)** The transition and transversion plot (Ti/Tv) showed the distribution of single nucleotide variation (SNV) in LAC-TB with a stacked bar plot at the bottom displaying the mutational spectrum distribution of each sample.


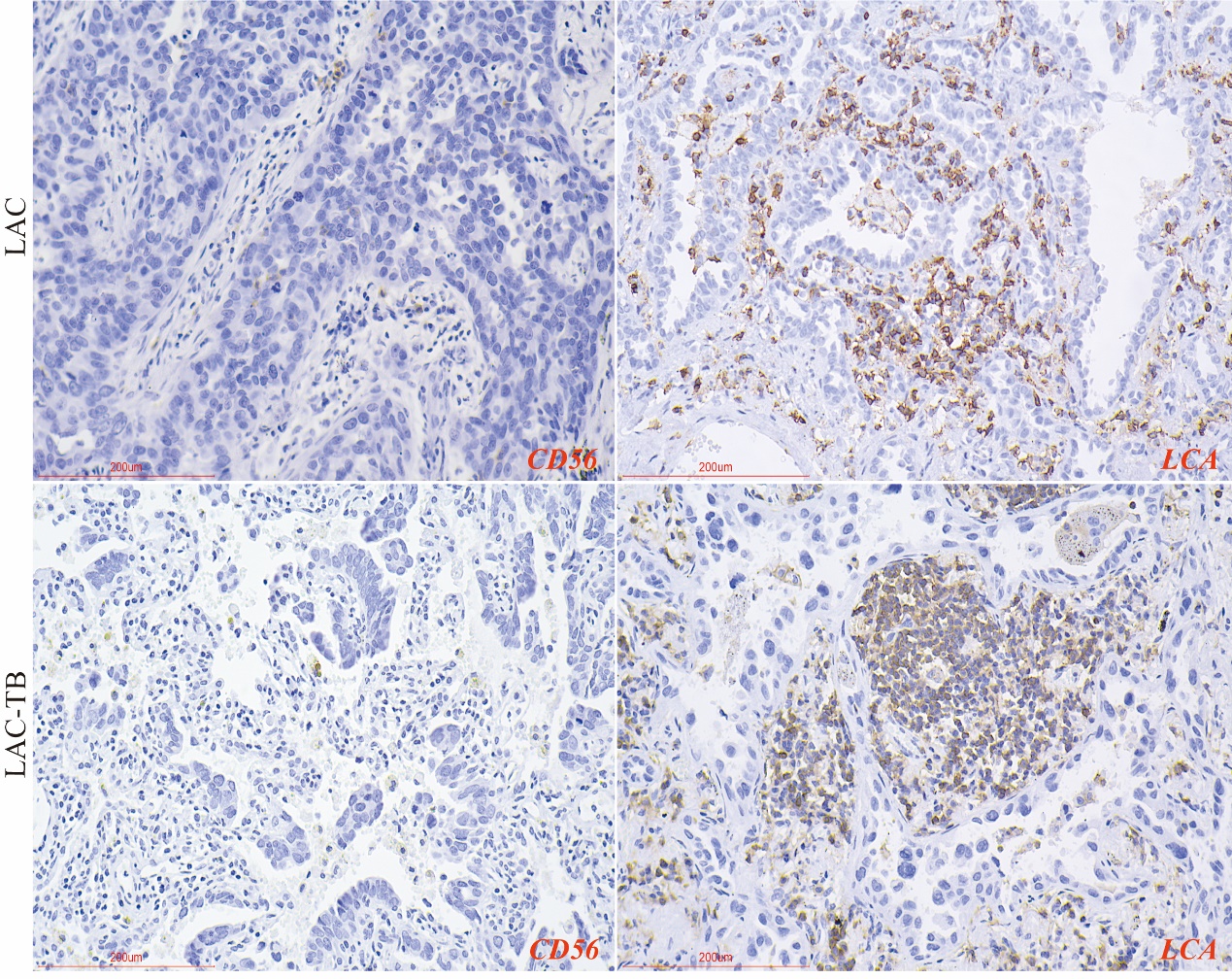


**Supplementary Figure 3.** The immunohistochemical staining of immune cell markers CD56 and LCA in the tumor area of the LAC group and LAC-TB group. Immunohistochemical staining (200×), Scale bar: 200 µm.

## Supplementary Tables

## Supplementary Table 1 Comparison of positive rates of expression of LAC markers between the LAC-TB and LAC groups

| Markers | positive rates of expression (%, n) | | *P* |
| --- | --- | --- | --- |
|  | LAC | LAC-TB |  |
| TTF-1 | 100 (14) | 100 (14) | / |
| Napsin A | 100 (14) | 100 (14) | / |
| CK | 100 (14) | 100 (14) | / |
| CK7 | 100 (14) | 100 (14) | / |
| CD56 | 1(14) | 1(14) | / |
| P40 | 0(14) | 0(14) | / |
| CK5/6 | 0(14) | 0(14) | / |

## Supplementary Table 2 Comparison of a Ki-67 proliferation index between the LAC-TB and LAC groups

| Type | a Ki-67 proliferation index (%, n) | | *P* |
| --- | --- | --- | --- |
|  | ≥10% | <10% |  |
| LAC | 21.43 (3) | 78.57 (11) | 0.021 |
| LAC-TB | 71.43(10) | 28.57 (4) |  |

## Supplementary Table 3 Comparison of the expression of LAG-3 on tumor cells between the LAC-TB and LAC groups

| Type | LAG-3^+^Tumor Cell（%, n） | | | *P* |
| --- | --- | --- | --- | --- |
|  | Negative | Low expression | High expression |  |
| LAC | 21.43（3） | 71.43（10） | 7.14（1） | 0.272 |
| LAC-TB | 28.57（4） | 42.86（6） | 28.57（4） |  |
